# Assessing returns to research investments in rice varietal development: Evidence from the Philippines and Bangladesh

**DOI:** 10.1016/j.gfs.2022.100646

**Published:** 2022-06

**Authors:** Rowell C. Dikitanan, Valerien O. Pede, Roderick M. Rejesus, Humnath Bhandari, G.M. Monirul Alam, Robert S. Andrade

**Affiliations:** a Impact Evaluation, Policy and Foresight Unit, International Rice Research Institute, Los Banos, Philippines; b Impact Evaluation, Policy and Foresight Unit, International Rice Research Institute, Los Banos, Philippines; c Department of Agricultural and Resource Economics North Carolina State University, North Carolina, USA; d Impact Evaluation, Policy and Foresight Unit, International Rice Research Institute, Dhaka, Bangladesh; e Department of Agribusiness, Bangabandhu Sheikh Mujibur Rahman Agricultural University, Gazipur, Bangladesh; f Foresight and Applied Economics for Impact, Alliance of Biodiversity International and CIAT, Cali, Colombia

## Abstract

• This study estimates economic returns to investments in rice varietal development in the Philippines and Bangladesh.
• The net returns to IRRI and national partners' investments remain strongly positive.
• However, the returns are decreasing at a faster rate in the Philippines (24%) than in Bangladesh (6%).
• IRRI and national partners should continue investing in rice R&D, especially to develop superior rice varieties.

## 1 Introduction

Research and development (R&D) investments in agriculture have long been used by rice-growing nations in Asia to boost or maintain production, help overcome domestic food insecurity, and meet economic development goals. For the past half century, national and international research organizations such as the Consultative Group on International Agricultural Research (CGIAR) have mobilized and invested large amounts of resources oriented to advancing further rice R&D and strengthening national research partners. For example, there is a long history of collaborative research between the International Rice Research Institute (IRRI, a founding member of the CGIAR), and national agricultural research and extension system (NARES) partners to address rice production challenges throughout R&D that can produce improved rice varieties, develop efficient agronomic practices and technologies, and increase the scientific capacity of local researchers. Although agricultural R&D investment is increasing over time, public spending in agricultural R&D is lower in developing countries than in developed countries, and the main countries investing have shuffled over time, with a substantial growth for middle-income countries (Beintema and Stads, 2008; Pardey et al., 2018). In the Philippines and Bangladesh, for instance, only 0.4% of their agricultural gross domestic product (GDP) was invested in agricultural research in 2017 and 2016, respectively (Stads et al., 2019, 2020).

A relatively large investment in rice R&D compared to other crops and the subsequent adoption of improved varieties^1^ produced by these investments have arguably benefited rice production growth in several developing countries in Asia, such as the Philippines and Bangladesh (Hossain et al., 2006). Improved varieties in the Green Revolution era of the 1960s through the 1980s have arguably been the main research innovation that has driven rice production growth in Asia (Hossain et al., 2003; Pingali, 2012; John and Babu, 2021). By the late 1990s, improved inbred and hybrid varieties of rice had replaced the most commonly adopted varieties on more than 100 million harvested hectares annually in Asia. Hossain et al. (2003) suggested that the annual gains from the improved varieties of rice adopted in South and Southeast Asia were USD 10.8 billion (nominal) per year in the late 1990s. As such, R&D investments from IRRI and its NARES partners are often mentioned as one of the factors that have contributed to the robust rice production growth in Asia through the 1990s.

However, questions have been raised whether rice varietal improvements continue to be a relevant factor that influences rice productivity growth in the major rice-producing nations in Asia, especially in more recent years. For example, several articles have indicated that the pace of varietal replacement in many parts of Asia has slowed down and there have been smaller increases in yield potential since the 1990s (Wang et al., 2012). Some have even implied that rice yield growth has started to fall below rice consumption growth such that future food security might be compromised if this trend continues (Mohanty et al., 2010). Therefore, it is an appropriate time to assess whether recent rice research investments by IRRI and its NARES partners in varietal development programs still generate commensurate positive net economic returns in terms of rice production growth in the major rice-producing countries in Asia.

The objective of this study is to evaluate the net economic contributions of IRRI's rice varietal improvement efforts with regard to rice production growth in two major rice economies of Asia, namely, the Philippines and Bangladesh. Specifically, we examine whether the value of rice production growth due to IRRI and its NARES partners' varietal development programs outweighs the investments made in these programs for the Asian countries. Data from a variety of sources were used to operationalize the economic surplus approach of Alston et al. (1998) and determine the net economic returns to rice research and extension investments in IRRI's varietal improvement programs.

## 2 Background on rice varietal development

### 2.1 Philippines

In the Philippines, rice germplasm produced or conserved by IRRI is made freely available to domestic rice breeding institutions (e.g., the Philippine Rice Research Institute (PhilRice), the University of the Philippines at Los Baños (UPLB) the Bureau of Plant Industry (BPI), and private companies) (Launio et al., 2008). Varieties released from 1965 to 1975 are identified as first-generation improved varieties, which includes the IR series^2^ of varieties (*IR5* to *IR34* developed by IRRI) and the C4 series of varieties (developed by UPLB). Under ideal conditions, the first-generation improved varieties have higher yield potential than those commonly adopted at that time. However, this performance differential is not necessarily observed in farmers' fields because of the improved varieties’ susceptibility to pests and diseases (Hossain and Pingali, 1998).

Estudillo and Otsuka (2006) reported that the significant yield increases seen in irrigated ecosystems up until 1997 can largely be attributed to improved rice varieties. They indicated that the major yield gains in the production regions of the Philippines were made possible due to the second-generation improved rice varieties, consisting of *IR36* to *IR62* (which were released from 1975 to 1984). These varieties had improved resistance to major pests and diseases (as compared to the first-generation improved varieties, for which the focus was on higher yield potential and higher input requirements).

The third-generation improved varieties consisted of *IR64* to *IR72* and *PSB Rc 2* to *PSB Rc 74*, which were released from 1985 to 1995. These varieties incorporated better grain quality and stronger host-plant resistance in the rice plants (Launio et al., 2008). The fourth-generation improved varieties were varieties released after 1995, which targeted more difficult production environments (i.e., drought-prone environments).

**Fig. 1:**
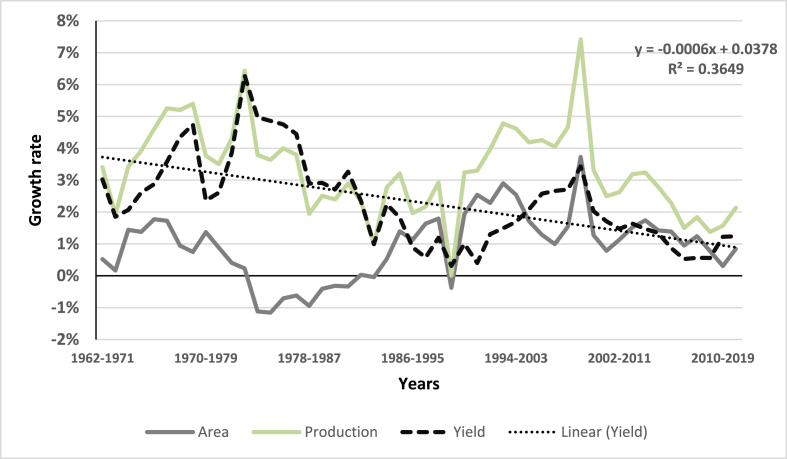
Ten-year moving average growth rate of area, production, and yield of rice in the Philippines, 1990–2018. Source: Food and Agriculture Organization of the United Nations (2020).

Over the past five decades, many new improved varieties have been released in the Philippines (see Appendix Table A1). Based on information from the National Seed Quality Control Services of BPI, a total of 168 rice varieties were grown in the Philippines for the period 1990 to 2018. The adoption pattern of the leading varieties is shown in Appendix Figure A1. In the 1990s, *IR72* and *IR64* were the popular varieties. But, over the past decade, the more popular newer varieties used in the Philippines include *NSIC Rc222* and *PSB Rc18,* although older varieties are still adopted mainly because of specific consumer preferences. Nonetheless, the share of IRRI genetic materials planted has diminished over time, similar to the general trend of the 10-year moving average growth rate of yield that has diminished as well over time, but it has remained positive for the past three decades (Fig. 1). Average yields from the 1990s (2.87 t/ha) have increased almost by a ton compared to the last decade average of 3.9 t/ha.

### 2.2 Bangladesh

Most of the rice varieties released in Bangladesh are from the Bangladesh Rice Research Institute (BRRI) and the Bangladesh Institute of Nuclear Agriculture (BINA), where IRRI germplasm is mainly used in the breeding programs. IRRI, directly or indirectly, has contributed to the development of 87 out of the 89 leading improved rice varieties grown in Bangladesh for the period 1990 to 2018 (see Appendix Table A2). Before Bangladesh's independence in 1971, the first IRRI-bred improved variety introduced in the country was *IR8* for the *Boro*^3^ season (Hossain et al., 2006). In 1970, the IRRI-bred variety *IR20* was introduced in Bangladesh for cultivation in the *Aman*^4^ season (Hossain et al., 2006). Since 1973, both BRRI and IRRI have cooperatively engaged in adaptive research to evaluate rice germplasm and release new improved varieties suited for the country. From the late 1970s to the early 1990s, many new varieties were developed and released primarily to overcome susceptibility to insects and diseases, but not necessarily for higher yield per se. Only *BRRI dhan29*, which was released in 1994, surpassed *BR3* (released in 1973) in terms of average yield. In addition, many improved varieties released in the 1990s had shorter plant height, better grain quality, and a shorter maturity period than the varieties released in the 1970s.

**Fig. 2:**
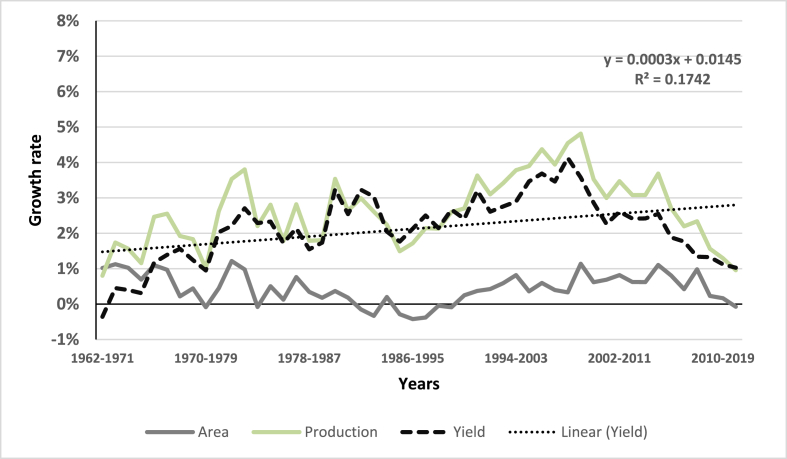
Ten-year moving average growth rate of area, production, and yield of rice in Bangladesh, 1990–2018. Source: Food and Agriculture Organization of the United Nations (2020).

Appendix Figure A2.1 presents the adoption patterns of the leading rice varieties in the *Boro* season. Note that varieties *BRRI dhan28* and *BRRI dhan29* (released in 1994) have dominated rice production in the *Boro* season in Bangladesh over the past 20 years. On the other hand, *BR11* and *BRRI dhan49* have dominated rice production in the *Aman* season in the past 20 years. It seems that recently developed varieties have not yet been widely adopted in Bangladesh (at least for the *Boro* season). However, this trend is not observed in the *Aman* and *Aus*^5^ seasons, since a larger mix of varieties has been adopted in more recent years (see Appendix Figures A2.2 and A2.3). With the patterns of rice variety adoption observed in Bangladesh over time, it is not surprising that average rice yields have increased substantially since the 1970s (Fig. 2). The average rice yield increased 2.6 times over the four decades from 1.75 t/ha in the 1970s to 4.57 t/ha in the past decade. The significant increase in rice production, the staple food, has transformed Bangladesh from a food-deficit into a food self-sufficient country, despite the more than 2.5 times increase in population. However, the 10-year moving average growth rate has decreased lately for average yields.

## 3 Calculating rates of return

This study uses an economic surplus approach to calculate the flow of benefits due to uptake of improved rice varieties, and then compares them with the R&D and extension cost flow required to develop the improved rice varieties. This allows us to empirically estimate rates of return (NPV: net present value, BCR: benefit-cost ratio, IRR: internal rate of return, and MIRR: modified internal rate of return) to IRRI and its NARES partners' investment in rice varietal development in the Philippines and Bangladesh.

### 3.1 Benefits calculation

#### 3.1.1 Economic surplus model

This method has been used to evaluate the welfare effects of new agricultural technologies and research programs within a partial equilibrium framework (Alston et al., 1998; Brennan and Malabayabas, 2011; Rejesus et al., 2014; Labarta et al., 2017; Schreinemachers et al., 2017; Shew et al., 2019; Sequeros et al., 2020). Although the impacts of rice R&D innovations in CGIAR programs have been largely examined in the economic literature through ex post assessment methodologies (see Yamano et al., 2016; Mishra et al., 2022 for recent reviews) and through ex ante assessment of new and future technologies, the economic surplus approach offers the advantage that it allows revealing more aggregated benefits over longer time spans. Reviews by Hurley et al. (2016) focusing on 492 separate studies published from 1958 to 2015 and by Pardey et al. (2016) focusing on 113 studies published from 1975 to 2014 spanning 25 countries indicate that the economic surplus approach remains relevant in the economic literature to examine the returns on R&D investment, although new approaches, mostly econometric-based, are now being used. For instance, Yigezu et al. (2021) recently estimated the returns to national and international investments in wheat research for Morocco using the endogenously switching regression (ESR) model applied to a nationally representative sample survey of 2296 wheat fields and cost estimates from public and CGIAR investments on wheat research in Morocco. Although the econometric approaches appear to be less restrictive than the Alston et al. (1998) surplus approach in terms of assumptions, they have the disadvantage of relying on survey data from a shorter time span, and often cannot appropriately generate aggregated returns at the country or regional level. The Alston et al. (1998) approach remains appealing for its simplicity, it has benefited from improvement over time based on criticisms of earlier applications, and we justify our parameter selection based on the best empirical information available, which in turn strengthens our calculations.

We followed the Alston et al. (1998) surplus model, which measures a shift in output supply caused by technology or research (i.e., in our case, a supply shift due to the adoption of new rice varieties developed by IRRI) and estimates the net welfare effects through the resulting changes in economic surplus. To strengthen estimation of the economic surplus measures (see Alston et al., 1998), we comprehensively describe our assumptions. For instance, with regard to the within-country economy/trade contexts and the elasticity of rice demand, we assume a small open-economy context for the two countries in the study. This assumption also suggests that the countries are “small” enough such that changes in production levels within the country do not influence world rice prices (i.e., prices remain constant). This further implies that the demand curve is perfectly elastic (i.e., flat) and welfare benefits due to a technology or varietal-research-induced supply shift accrue to producers (i.e., they increase producer surplus but do not affect consumer surplus). We also assume that a supply shift is parallel rather than pivotal. This is consistent with the recommendation of Alston et al. (1998) and this assumption has been used in several economic surplus studies for rice technologies to date (see Alpuerto et al., 2009; Brennan and Malabayabas, 2011). However, Alston et al. (1998) also pointed out that, with a linear supply curve, the total benefits from a parallel shift are almost twice the size of the total benefits from a pivotal shift. Given this insight, a pivotal shift effect ‒ calculated as half of the parallel shift effect ‒ can serve as a lower bound estimate. Further, conflation issues mentioned by Raitzer et al. (2015) for the methodology were not addressed due to data limitations.

**Fig. 3:**
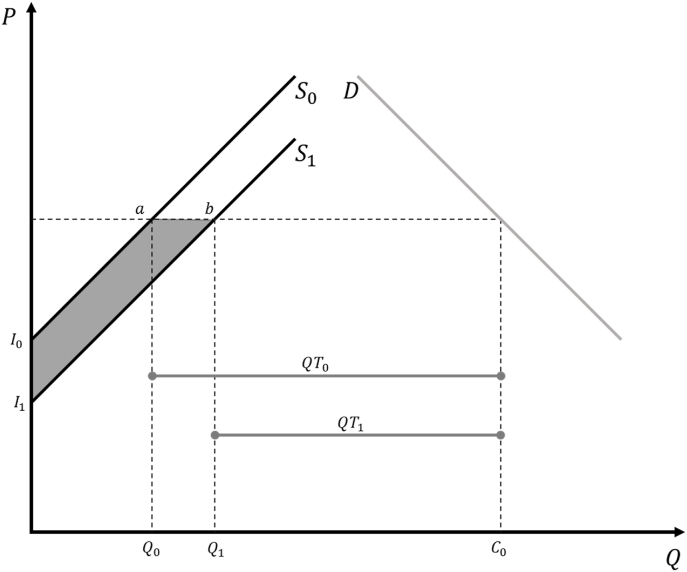
Economic surplus model for small open economy. (Alston et al., 1998, page 227).

Fig. 3 presents a graphical representation of the upgraded economic surplus model implemented in this study (i.e., where the above assumptions are imposed). The initial equilibrium is defined at the following point: consumption at $C_{0}$, domestic production at $Q_{0}$, world rice price at $P_{W}$, and net imports (i.e., the difference between $Q_{0}$ and $C_{0}$) equal to $QT_{0}$. A research intervention (e.g., new higher-yielding variety) is assumed to result in a parallel shift of the supply curve from $S_{0}$ to $S_{1}$, which results in lower rice importation, $QT_{1}$. With a small open-economy assumption (and constant $P_{W}$), the total change in economic surplus, $I_{0}abI_{1}$, is all producer surplus.

The algebraic formulas to calculate changes in the total economic surplus (which is equivalent to the change in producer surplus in this case) are as follows:

(1) ΔCS=0

(2) $$\Delta PS\;=\;\Delta TS\;=P_{w}Q_{o}K\left(1+0.5K\varepsilon \right)$$

where ΔCS is the change in consumer surplus, ΔPS is the change in producer surplus, ΔTS is the change in total surplus, $P_{w}$ is the constant rice price, $Q_{o}$ is the pre-intervention production level, K is the so-called K shift parameter that represents the vertical shift in supply (expressed as a proportionate cost decrease per ton due to the intervention), and ε is the supply elasticity of rice in the country. The K shift parameter is calculated as

(3) $$K_{t}\;=\;\left[\frac{E\left(Y\right)}{\varepsilon }-\frac{E\left(C\right)}{1-E\left(Y\right)}\right]\rho A_{t}\left(1-\delta_{t}\right)$$

where E(Y) is the expected proportionate yield change per hectare due to the research intervention, ε is the supply elasticity of rice in the country, E(C) is the proportionate change in input costs per hectare (if any), ρ is the probability that the new technology will fully achieve the yield change E(Y), $A_{t}$ is the rate of adoption in year *t*, and $\delta_{t}$ is the rate of annual depreciation of the new research intervention (Alston et al., 1998. Chapter 5, page 360).

#### 3.1.2 Definition of parameters

To operationalize the economic surplus model, we estimate the expected proportionate rice yield change per hectare E(Y) between those IRRI/NARES-related varieties and those that were non-related. We contrast the improved varieties developed by IRRI and its NARES partners in the Philippines and Bangladesh with those non-related to IRRI or NARES breeding programs. This requires information on (1) the average yield changes due to the adoption of different rice varieties in a particular country over the years and (2) the proportion of those average yield changes that can be attributed to IRRI or partner NARES research over the years.

Also, we first assess the adoption $A_{t}$ and E(Y) yield impacts of the different improved varieties of rice used in the Philippines and Bangladesh. This requires information on the area planted to each rice variety available in both countries and the corresponding average attainable yields of these varieties. We obtained these data for the two countries from national databases of the BPI, PhilRice, BRRI, BINA, and Bangladesh Department of Agricultural Extension (DAE). For the Philippines, we build on the dataset used by Brennan and Malabayabas (2011), which has information on percentage of area planted from 1990 to 2009 and average attainable yields. Adoption rates from 2011 to 2018 were collected from BPI. For Bangladesh, BRRI annually collects data on adoption of BRRI-released rice varieties through sample household surveys. In addition, DAE annually collects data on adoption of major rice varieties through district surveys. For non-BRRI varieties, the data on area planted to each variety were collected from DAE. The data on variety-specific yields were collected from BRRI, BINA, and DAE. For both the Philippines and Bangladesh, the data on area planted and yield were not available for some varieties for some years. The data on area planted were generated based on interpolation and the data on yields were generated based on national average yields based on Food and Agriculture Organization of the United Nations (FAO) and Bangladesh Bureau of Statistics (BBS).

Using the information on area planted and variety-specific yields, we then calculated the area share between IRRI and non-IRRI varieties. This requires determining how much IRRI contributed to the development of each variety. This allows us to generate a proxy for the IRRI and partners’ contribution to the proportionate yield changes observed in each country. However, calculating the proportion attributable only to IRRI for each rice variety adopted is inherently complex since most successful research and extension is a collaborative process (Alston and Pardey, 2001).

Given the attribution constraints, two approaches are applied from previous studies: the “last cross rule” and the “geometric rule.” According to Pardey et al. (2002), the last cross rule gives 100% credit for a particular variety to the breeder who produced it and none to its parents that still exist as varieties in their own right. On the other hand, the geometric rule uses a geometrically declining set of weights, mimicking somewhat the share of genetic material carried forward from earlier nodes in the pedigree into the present variety according to Mendel's law of heredity. In our calculation, we use the geometric rule up to the immediate parents' level. The geometric rule attribution method has been used by Hossain et al. (2003) and Brennan and Malabayabas (2011), and it allows us to calculate rates of return under the two rules, which provides a range of estimates for possible attribution.

Parentage information to implement the IRRI attribution rules was gathered primarily from the International Rice Information System (IRIS) variety database, BRRI, and BINA to enable calculation of the IRRI contributions to each of the rice varieties grown in the Philippines and Bangladesh for the time period considered. Based on the adoption rate between IRRI + partners' and non-IRRI + partners' varieties using the attribution rules described above, we can then calculate the expected proportionate change in yield per hectare due to IRRI and its NARES partners’ varietal improvement research and extension.

The K shift parameter represents the unitary-cost change that uses information about the following parameters: the supply elasticity of rice in the country, the proportionate change in input costs per hectare, the probability that the new variety will fully achieve the estimated change in yield, the rate of adoption of the variety in year *t*, and the rate of annual depreciation of the new research intervention according to equation (3). Note that we conservatively assumed that adoption of new varieties requires a consequent increase in input costs by 2% (Hossain and Bayes, 2018) and yield effects of new varieties depreciate by 10% based on expert opinions.

For the Philippines, we assumed a rice supply elasticity of 0.28, which was the mean value based on three supply elasticities: 0.15, 0.40, and 0.30, as reported by Mohanty et al. (2010), Alpuerto et al. (2009), and Mamaril (2002), respectively. For Bangladesh, we chose a rice supply elasticity of 0.25 because the literature reports rice supply elasticities for Bangladesh in the range of 0.20–0.30 (Ahmed, 1997; Dorosh and Rashid, 2012). Moreover, for both countries of interest, we do not require the probability of success because we are calculating an ex post scenario.

After the K shift parameter is calculated, the yearly change in total surplus can be computed (using information on prices, pre-intervention production levels, and the K shift parameter described in equation (2). National rice production levels and rice price data (for which 1990 is the base) were collected from the World Rice Statistics (WRS) database for both the Philippines and Bangladesh. The Consumer Price Index data used to deflate/inflate rice producer prices and research and extension costs are also available from the WRS database.

### 3.2 R&D and extension cost calculation

After the yearly total surplus changes are calculated, the values can be compared to the yearly IRRI and NARES partners' investment costs to calculate a yearly net surplus change, and therefore rates of return (i.e., yearly investment costs are subtracted from the estimated yearly total change in surplus). We use annual research and extension cost data available from IRRI's Portfolio Management Office and Agricultural Science and Technology Indicators (ASTI) of the International Food Policy Research Institute to calculate the yearly investment costs for the study period from 1990 to 2018. For IRRI investments, equal grant allocation per country and per year was assumed in computing IRRI costs (including management cost) allocated to the Philippines^6^ and Bangladesh. We conservatively assumed that government R&D investments on rice were 30% and 20% of the national agricultural research expenditure for the Philippines and Bangladesh, respectively, based on the crop of interest given by the full-time equivalent allocated to each crop that corresponds to that share (ASTI, 2022; Gert-Jan et al., 2007; World Bank, 2020). The complementary extension cost was based on the budget of the Philippines Department of Agriculture that estimates that the rice research cost is roughly the same as the extension cost for the Philippines (World Bank, 2020). For Bangladesh, to define the extension cost, we based our estimate on direct interviews with scientists from BRRI and the DAE on rice R&D in Bangladesh, who considered that the extension cost was also estimated as roughly equivalent to the cost of R&D investment. Finally, we include national agricultural research expenditure data from ASTI, even though it is limited to 2002 for the Philippines and 2000–2016 for Bangladesh. Missing data for both countries were forecasted using the country's GDP (World Bank, 2022) and an expected share of those resources devoted to rice R&D. An initial R&D investment of an extra five initial years is added in the total investment costs to incorporate past investments.

### 3.3 Rates of return

Finally, we use the most common rates of return. We assume a reasonable discount rate (i.e., 5% in this case) commonly used in surplus analysis (Brennan and Malabayabas, 2011; Rejesus et al., 2014; Raitzer et al., 2015). The yearly net surplus changes can then be summed together over the period under consideration in this study (1990–2018) to obtain the net present value (NPV) of the net returns from IRRI and NARES investments in varietal development, and this was calculated as

(4) $$NPV\;=\;\overset{T}{\underset{t=0}{\sum }}\;\frac{B_{t}\;-C_{t}}{1\;+\;r^{t}}$$

where $B_{t}$ represents the benefits at the time *t*, $C_{t}$ represents investment and recurrent cost at time *t*, *T* represents the period under consideration, and *r* represents the discount rate (for the IRR and MIRR, $r^{t}$ represents the discount rate, while $r^{b}$ represents the reinvestment discount rate and $r^{c}$ represents the borrowing discount rate). Other indicators such as benefit-cost ratio, internal rate of return, and modified internal rate of return are also calculated to evaluate the investments made in IRRI and national breeding programs. The BCR is calculated as

(5) $$BCR\;=\;\overset{T}{\underset{t=0}{\sum }}\;\frac{B_{t}}{1\;+\;r^{t}}/\overset{T}{\underset{t=0}{\sum }}\;\frac{C_{t}}{1\;+r^{t}}$$

Solving equation (6) below will yield the IRR:

(6) $$0\;=\;\overset{T}{\underset{t=0}{\sum }}\;\frac{B_{t}\;-\;C_{t}}{1\;+\;IRR^{t}}$$

Finally, we use the MIRR given by

(7) $$MIRR\;=\;\sqrt[{T}]{\frac{\sum_{t=0}^{T}\;B_{t}{\left(1\;+\;r^{b}\right)}^{T-t}}{\sum_{t=0}^{T}\;C_{t}{\left(1\;+\;r^{c}\right)}^{-t}}}-\;1$$

## 4 Results and discussion

### 4.1 Returns to IRRI and NARES’ partners for varietal investments in the Philippines

**Fig. 4:**
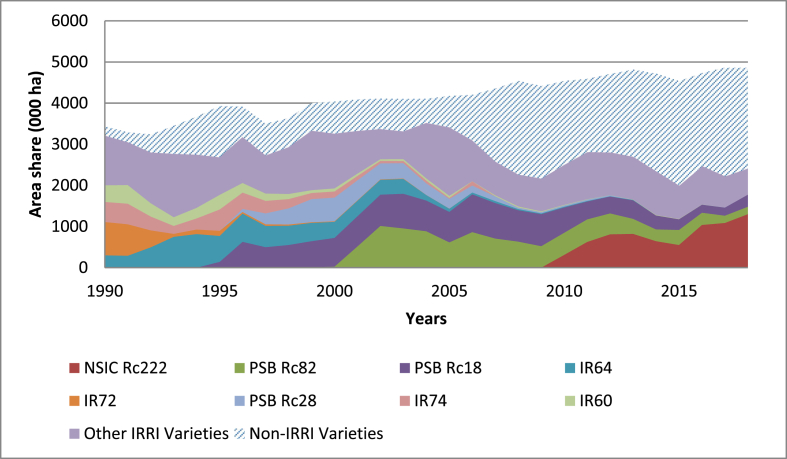
Area (000 ha) share between IRRI and non-IRRI varieties in the Philippines based on the last cross rule, 1990 to 2018.

**Fig. 5:**
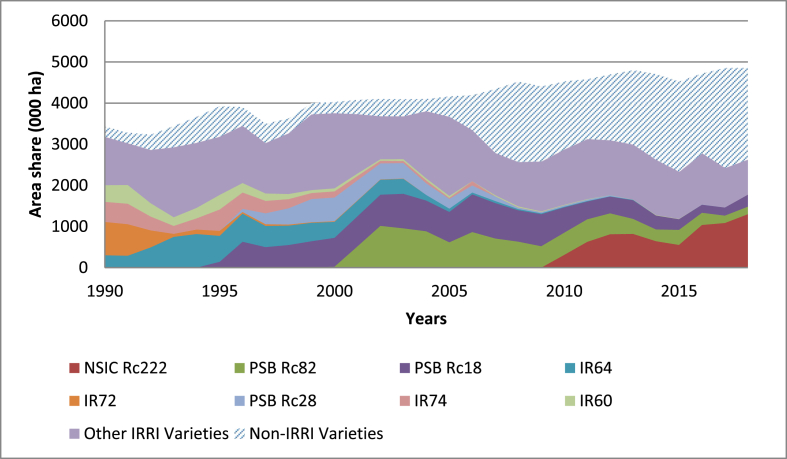
Area (000 ha) share between IRRI and non-IRRI varieties in the Philippines based on the geometric rule, 1990 to 2018.

Fig. 4, Fig. 5 show that IRRI contributions to the varietal mix used in the Philippines is trending slightly downward. This suggests that most varieties used in recent years tend to have fewer IRRI attributes than the varieties used in the 1990s. This is primarily because NARES are able to breed and disseminate more rice varieties themselves, which was the objective of CGIAR since its inception: to strengthen local partners. IRRI has a long history of providing technical and capacity-building support to the NARES to develop their own rice varieties in multiple countries around the world.

Using constant producer prices (normalized to 1990 dollars), yearly production, and the necessary parameters from the conceptual model, the benefits flow generated from the varietal improvements of IRRI together with the NARES contribution (or the total surplus contributions of IRRI and partners' varietal development efforts) can be estimated. Subtracting the yearly R&D and extension investment costs from these yearly total surplus values (assuming a 5% discount rate), we estimate that the NPV of IRRI's contributions to varietal yield changes in the Philippines for the 1990 to 2018 period is approximately USD 4.24 billion and USD 3.61 billion based on the last cross and geometric attribution rule, respectively. The corresponding overall BCR is about 9:1 and 7:1 for IRRI and its NARES partners' investment in varietal yield improvement in the Philippines. The IRR is 54% and 49% based on the last cross and geometric attribution rule, respectively. The MIRR is about 13% based on both attribution rules. Overall, these figures suggest that there are still positive net payoffs to IRRI and its NARES partners' research investments in breeding new rice varieties in the Philippines for the period 1990 to 2018. Appendix Figure A3.1 shows the discounted total benefits and costs in the Philippines based on the two attribution rules over the period 1990–2018. It can be noted that the returns to IRRI varietal research investments have been decreasing by 24%, on average.

### 4.2 Returns to IRRI varietal investments in Bangladesh

**Fig. 6:**
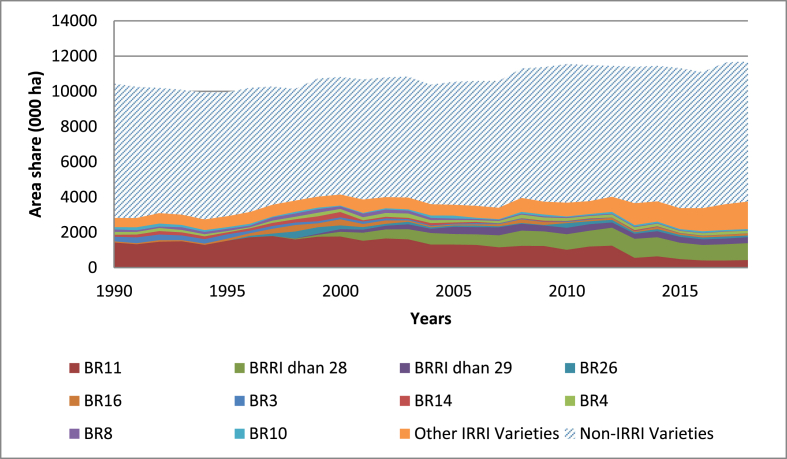
Area (000 ha) share between IRRI and non-IRRI varieties in Bangladesh based on the geometric rule, 1990 to 2018.

All varieties in Bangladesh used during the period of analysis were developed by the local partners; hence, only the geometric attribution rule applies. IRRI contributions to the varieties used in the country from 1990 to 2018 are presented in Fig. 6. Note that it is an important achievement for the local partners to become the main source for the final breeding varieties, while IRRI remains as the source for basic germplasm. Regarding the proportion contributed by research, we see that IRRI's contribution is trending upward vis-à-vis the downward trend for the Philippine case. This is because the local partners rely on the original germplasm provided by IRRI or advanced lines provided from IRRI's breeding program. However, the share between IRRI and non-IRRI varieties in Bangladesh tends to be lower than for the Philippines under the geometric rule, which offers an opportunity to continue providing the basic research needed to generate new varieties.

Using Bangladesh-specific information for all the parameters in the conceptual model, we calculate the year-to-year total surplus contributions of IRRI and partners' varietal development efforts for the 1990 to 2018 period. Assuming a 5% discount rate, we estimate that the NPV of IRRI's and its partners' contribution to varietal yield changes for the 1990 to 2018 period in Bangladesh is approximately USD 33.32 billion. The corresponding overall BCR is about 115:1 for IRRI's and its NARES partners' investment in varietal yield improvement in Bangladesh. The IRR and MIRR are about 179% and 26%, respectively. In general, the estimated net returns from IRRI varietal development investments in Bangladesh are still substantial, given that R&D investments are low but the yield differences between IRRI and non-IRRI varieties are quite large. Similar to our findings for the Philippine case, returns to IRRI varietal research investments are decreasing in recent years but at a lower decreasing rate of 6%, on average, as shown in Appendix Figure A3.2.

### 4.3 Discussion

Our study provides evidence that the value of rice production growth due to IRRI and partners' varietal development programs outweighs the investments made in these programs by far. In other words, investments made in varietal improvement still generate positive net economic returns. This finding reinforces the already recognized importance of rice and its profitability as an investment that contributes to the rice value chain.

This study contributes to the literature by providing new empirical estimates of the returns to IRRI and its partners' investments in varietal improvement for the period 1990 to 2018. Previous studies have provided estimates for earlier or overlapping periods. For example, Brennan and Malabayabas (2011) indicated that the NPV of the returns to IRRI's rice varietal improvement outputs in the Philippines for the period 1985 to 2009 was approximately USD 4.3 billion (in 2005 USD), while Raitzer et al. (2015) suggested that, from 1989 to 2009, the NPV of the returns to IRRI investments in developing rice varieties in the Philippines was about USD 1.2 billion (in 2005 USD). Similarly, Raitzer et al. (2015) estimated that IRRI's contribution to the development of improved rice varieties in Bangladesh was about USD 4 billion (in 2005 USD) over the 1989 to 2009 period studied. Given that the base years in these studies are not the same as ours (with the focus countries not being the same) and the time period is different across studies, a direct comparison of the NPV is not realistic, but we can still highlight the economic importance of the activity and the R&D returns generated.

Comparisons are made on the other metrics used, such as BCR and IRR. Table 1 shows estimates of BCR and IRR from several global studies, and some specific for rice R&D investment, and includes at the end of the table our estimates in contrast with others. Our estimates for BCR in the Philippines are within the same magnitude as those obtained by Alston et al. (2021), Hurley et al. (2016), and Pardey et al. (2016). Although our estimates of BCR for Bangladesh are higher than most of the median values reported in other studies, they are still within the distribution of rates of return, specifically in the fourth quartile of the global distribution shown by Hurley et al. (2016). The same situation is observed with IRR. In Bangladesh, the IRR is located in the fourth quartile of the global distribution, while the MIRR becomes more conservative given its specific attributes to calculate.

**Table 1:** Estimated benefits from research on rice genetic improvement.

Studies	Study period	BCR	IRR (%)	Countries
Alston et al. (2021)	1958–2020	7^a^	–	Global^b^
		12^c^		Global^b^
		9^d^		Global^b^
Hurley et al. (2016)	1958–2015	12^e^	38^e^	Global^f^
			53^g^	
Pardey et al. (2016)	1975–2014	11^e^	35^e^	Sub-Saharan Africa^h^
			75^g^	
Brennan and Malabayabas (2011)	1985–2009	22	28	Indonesia, Philippines, and Vietnam
Gautam (2009)	1997–2017	–	167	Eastern India
Jaroensathapornkul (2007)	1974–2000	7	–	Thailand
Fan et al. (2005)	2000	23^i^	–	China and India
This study	1990–2018	7^j^	49^j^	Philippines
		9^k^	54^k^	
		115^j^	179^j^	Bangladesh

a Median value for rice in CGIAR-related technologies.

b The study focused on nine separate studies published from 1958 to 2020.

c Median value for rice in non-CGIAR-related technologies.

d Conditional prediction for BCR for IRRI innovations.

e Rate of return to food and agriculture investment.

f The study focused on 36 separate studies published from 1958 to 2015.

g Rate of return to rice investment.

h The study focused on four separate studies published from 1975 to 2014.

i Computation done using IRRI's total budget.

j Based on the geometric rule.

k Based on the last cross rule.

A noteworthy finding from this study is that, although the investments made in varietal improvement continue to generate positive net economic returns over time, the surplus is declining in both countries. It is also interesting to note that the returns are decreasing at a faster rate in the Philippines than in Bangladesh. Specifically, the declining rate is 24% for the Philippines and 6% for Bangladesh, on average. Although these findings warrant further investigations in future studies, we can speculate that the continued strong use of IRRI genetic materials in the work conducted by domestic rice breeding institutions in Bangladesh and the larger yield gap still closing could be among the driving forces.

## 5 Limitations of the study

Notwithstanding our contributions to understanding the net economic surplus impact of IRRI and its NARES partners' R&D and extension investments in the Philippines and Bangladesh, it is important to recognize the limitations of the study and mention promising opportunities for future research. First, numerous studies have examined the impacts of rice innovations and R&D investment in CGIAR through ex post impact assessment (Yamano et al., 2016; Mishra et al., 2022). These models of ex post impact assessment often focus on individual innovations using a variety of econometric methods that are less restrictive in terms of assumptions than the economic surplus analysis used here. The economic surplus method was used in this study because it allows deriving aggregate benefits from specific innovations and comparing them to the R&D and extension investment made. However, the original Alston et al. (1998) model itself has some limitations, which has led to some improvement noted in the more recent literature (see Dar et al., 2013; Pradesha et al., 2019). The bulk of the literature on return to investment focuses on the monetary valuation of conceived benefits and rarely quantifies the co-benefits such as social, nutritional, or environmental consequences of technologies (Alston et al., 2021). One of the limitations of the Alston et al. (1998) model is that it relies on a single market partial equilibrium model. A computational general equilibrium (CGE) type of model capable of capturing the effects of rice productivity growth across all sectors of a country's economy could provide much more resolution on the impacts of rice R&D and extension investments, but it requires parameters that are often non-existent for all the other crops that are part of the whole economy. Other limitations of economic surplus analysis include data uncertainty or measurement errors in the parameters used to derive actual benefits and costs, which can bias the estimated rates of return.

Second, the analysis used in our study is based on the proposition that improved varieties have led to a permanent upward shift in yield potential over what would have occurred otherwise. Improved varieties can lead to a permanent shift to a higher yield potential or the gain can be a “one-off” increase that is eroded over time. Given this permanent shift assumption, the important question is the magnitude of that shift in yield potential and the consequent shift in the rice supply curve. If IRRI's rice breeding program were to cease, the advantages of its breeding lines would likely decline over time until, at some future point, the “with-IRRI” and “without-IRRI” yield would be equal. However, for the short run, the proposition that, with continued investment, IRRI breeding outputs result in a permanent upward shift in yield seems to most suitably reflect their impact on the rice industry.

Third, it is important to note that new rice varietal releases are usually accompanied by contemporaneous changes in agronomic management practices (e.g., complementary changes in fertilization, pest control, etc.). At the same time, rice varieties developed through IRRI breeding programs are sometimes not aiming to be “yield-enhancing,” but rather are aiming to be “maintenance research” such that the plant becomes more tolerant of abiotic stresses (e.g., droughts, floods) and more resistant to biotic stresses (e.g., pests and diseases). Hence, a true measure of the economic benefits of IRRI-derived germplasm will, in part at least, be a measure of not only the “yield-enhancing” and “maintenance research” traits of the improved germplasm but also the management technologies and practices, input use efficiencies, and enabling institutional structures accompanying the improved varieties. Such economic and non-economic benefits are complex and extremely difficult to quantify and separate out from the “yield-enhancing” effect of the germplasm per se. Therefore, these additional changes are not explicitly separated out in our study although some portions of these ancillary changes may be embodied in the assumed yield impacts of the new rice varieties released over time. It should be recognized, however, that these accompanying changes play an important role in the eventual welfare impacts of the new varieties. Future studies that can disentangle the economic benefits from the “yield-enhancing” traits, the “maintenance research” traits, and the complementary inputs would be an interesting new research direction.

Lastly, it is important to recognize that IRRI's contributions to the rice research infrastructure of the Philippines and Bangladesh do not only involve advances in breeding better rice varieties. Over the years, IRRI has also invested in scientific capacity-building programs for breeders and scientists in the two countries to enhance their ability to develop improved rice varieties and technologies, which is reflected in the adoption trends and highlights the fulfillment of the whole CGIAR objective to strengthen our partners. Moreover, national and regional agricultural policies have been implemented largely through the efforts of IRRI and its NARES partners. The economic benefits from IRRI's investments in scientific capacity building and championing rice policies would likely increase the surplus estimates in this study by several orders of magnitude. Further quantification of these capacity-building and rice policy championing efforts is left for future research.

## 6 Conclusions

Providing a relative magnitude of returns to investments is important for donors so that they can see whether payoffs still exist to funding this type of varietal improvement research. These return estimates can serve as justification for further research investments in the future as well as help guide future allocations (i.e., which countries and what type of varietal efforts to support and fund).

The findings from this study suggest that net returns to IRRI rice varietal development efforts are still strongly positive in the Philippines and Bangladesh. The yield benefits of using rice varieties with IRRI parentage in the two countries more than compensate for IRRI and its NARES partners’ investments to develop and disseminate IRRI-related varieties. The results suggest that returns to IRRI investments in the Philippines and Bangladesh have slowed down relative to returns observed prior to the 1990s. The findings suggest that the use of IRRI genetic materials is likely to be decreasing in the Philippines. Hence, the net welfare contributions of IRRI-related varieties in the Philippines tend to be lower than in Bangladesh.

Our results show that the Philippines and Bangladesh have made huge investments in the development of improved rice varieties over the past three decades and the economic returns to these investments in rice research were significant. A technology-led rice productivity growth through investment in R&D would generate substantial returns, which could contribute to improving the livelihoods of millions of rice farmers and consumers in these two major rice economies of Asia.

Although the national research capacity to develop new rice varieties has improved over time, public agricultural research spending is still low, along with insufficient research capacity to use modern rice breeding methods and tools. Also, the national systems lack access to a large pool of germplasm and capacity to analyze and use that germplasm. To address these constraints, the NARES partners need support from advanced research institutes such as IRRI and other 10.13039/501100015815CGIAR members. Therefore, IRRI/10.13039/501100015815CGIAR should continue working on rice R&D in these two countries with priority to support the NARES in capacity development, use of modern breeding methods and tools, application of advanced research, and access to rice germplasm. The results showed that the returns to IRRI and its NARES partners' investment in rice R&D and extension in these two countries are substantial, but have been declining. Therefore, IRRI management and donors should allocate adequate resources to continue IRRI and its NARES partners' rice R&D and extension programs in the Philippines and Bangladesh.

## Funding

This work was supported by the 10.13039/501100015815CGIAR Research Program on Rice (RICE CRP), the Accelerated Genetic Gain in Rice (AGGRi) Alliance project (Grant ID OPP1194925) and the CGIAR Initiative on Market Intelligence. No funding source had other involvement in the study.

## Declaration of competing interest

The authors declare that they have no known competing financial interests or personal relationships that could have appeared to influence the work reported in this paper.

## Footnotes

1 As suggested by one reviewer, we adopted the terminology “improved varieties” instead of “modern varieties” and also “most commonly adopted varieties at the time” instead of “traditional varieties” to avoid the common misconception about the connotations (modern = good or more productive; traditional = bad or less productive). In their article, Glover et al. (2019) discussed the misconceptions about improved technologies and the notions of technological change and adoption.

2 Rice crosses made at IRRI are assigned a number with IR (International Rice) as a prefix. These include semi-dwarf varieties of rice that were less likely to lodge (fall over).

3 *Boro* rice is the dry-season irrigated rice planted from December to early February and harvested from April to May. The development of groundwater irrigation systems and the incorporation of improved varieties accelerated the cultivation of *Boro* rice in Bangladesh.

4 *Aman* rice is the monsoon-season rainfed rice. It is planted in two ways: broadcasted in March or April after the *Boro* rice harvest in areas where water depth exceeds 0.5 m or transplanted from July to August in areas where water depth usually does not exceed 0.5 m. Both types are harvested from November through December. Broadcast *Aman* is grown mostly in the south and southeastern parts of the country whereas Transplanted *Aman* is grown throughout Bangladesh.

5 *Aus* rice is the pre-monsoon season rice generally grown under rainfed conditions as both a broadcast and transplanted crop scattered in most of the districts of Bangladesh. It is planted mostly in April–May and harvested in mid-July-August.

6 Including all basic research at IRRI headquarters.
