# Supplementary material for: Assessing returns to research investments in rice varietal development: Evidence from the Philippines and Bangladesh
Source: Glob Food Sec. 2022 Jun;33:100646. doi: 10.1016/j.gfs.2022.100646 (PMC9231557; doi:10.1016/j.gfs.2022.100646)
Supplement: Multimedia component 1 [file mmc1.docx]

**APPENDIX**

Appendix Table A1. IRRI’s contributions to rice varietal development in the Philippines, 1970-2018.

| Institution | Variety name | Generation^^[[1]](#footnote-1)^^ | Ave. Yield (t/ha) | Release year | Origin of variety^^[[2]](#footnote-2)^^ | Main Traits |
| --- | --- | --- | --- | --- | --- | --- |
|  |  |  |  |  |  |  |
| TV | Azucena | NA | 4.0 | 1956 | 4 | Aromatic |
| BPI | BPI 76 | 1 | 4.0 | 1959 | 4 | Moderate yield advantage |
| UPLB | C4-63G | 1 | 3.8 | 1968 | 4 | Moderate yield advantage |
| UPLB | C4-137 | 1 | 4.3 | 1969 | 4 | Moderate yield advantage |
| IRRI | IR22 | 1 | 4.28 | 1970 | 2 | Moderate yield advantage |
| UPLB | C-22 | 1 | 2.18 | 1972 | 4 | Moderate yield advantage |
| BPI | BPI 3-2 | 1 | 4.0 | 1973 | 4 | Moderate yield advantage |
| UPLB | C-168-134 | 1 | 4.03 | 1974 | 4 | Moderate yield advantage |
| BPI | BPI Ri-2 | 2 | 4.0 | 1975 | 2 | Moderate yield advantage |
| IRRI | IR29 | 2 | 3.74 | 1975 | 1 | Disease resistant |
| IRRI | IR32 | 2 | 4.47 | 1976 | 2 | Disease resistant |
| IRRI | IR36 | 2 | 4.86 | 1976 | 1 | Abiotic stress tolerant, Early maturing, Disease resistant |
| BPI | BPI Ri-4 | 2 | 5.06 | 1977 | 2 | Insect resistant, Disease resistant |
| IRRI | IR10 | 2 | 4.0 | 1977 | 4 | Moderate yield advantage |
| IRRI | IR42 | 2 | 5.04 | 1977 | 2 | Abiotic stress tolerant, Disease resistant |
| UPLB | UPL Ri-1 | 2 | 4.0 | 1977 | 2 | Sticky |
| IRRI | IR45 | 2 | 2.51 | 1978 | 1 | Abiotic stress tolerant, Disease resistant |
| IRRI | IR46 | 2 | 3.97 | 1978 | 1 | Abiotic stress tolerant |
| UPLB | UPL Ri-2 | 2 | 2.7 | 1978 | 4 | Insect resistant |
| BPI | BPI Ri-1 | 2 | 4.32 | 1979 | 4 | Disease tolerant |
| IRRI | IR50 | 2 | 4.5 | 1979 | 1 | Abiotic stress tolerant, Early maturing |
| IRRI | IR48 | 2 | 4.4 | 1980 | 1 | Abiotic stress tolerant, Disease resistant |
| IRRI | IR52 | 2 | 3.1 | 1980 | 1 | Abiotic stress tolerant, Disease resistant |
| IRRI | IR54 | 2 | 4.0 | 1980 | 1 | Disease resistant |
| UPLB | UPL Ri-5 | 2 | 2.68 | 1980 | 4 | Moderate yield advantage |
| BPI | BPI Ri-3 | 2 | 4.7 | 1981 | 4 | Disease resistant |
| UPLB | UPL Ri-7 | 2 | 3.04 | 1981 | 3 | Abiotic stress tolerant |
| IRRI | IR56 | 2 | 4.5 | 1982 | 1 | Abiotic stress tolerant, Early maturing, Insect resistant |
| UPLB | UPL Ri-4 | 2 | 4.7 | 1982 | 4 | Early maturing |
| BPI | BPI Ri-10 | 2 | 4.66 | 1983 | 4 | Disease resistant |
| IRRI | IR58 | 2 | 4.1 | 1983 | 1 | Abiotic stress tolerant, Early maturing, Disease resistant |
| IRRI | IR60 | 2 | 4.76 | 1983 | 1 | Abiotic stress tolerant, Early maturing, Insect resistant, Disease resistant |
| IRRI | IR62 | 2 | 4.77 | 1984 | 1 | Abiotic stress tolerant, Disease resistant |
| IRRI | IR64 | 3 | 5.31 | 1985 | 1 | Insect resistant, Disease resistant |
| IRRI | IR65 | 3 | 4.7 | 1985 | 1 | Disease resistant |
| BPI | BPI Ri-12 | 3 | 4.89 | 1987 | 2 | Sticky |
| IRRI | IR66 | 3 | 5.19 | 1987 | 1 | Early maturing, Insect tolerant, Disease tolerant |
| IRRI | IR68 | 3 | 4.48 | 1988 | 1 | Insect tolerant, Disease tolerant |
| IRRI | IR70 | 3 | 4.8 | 1988 | 1 | Abiotic stress tolerant, Insect resistant |
| IRRI | IR72 | 3 | 5.0 | 1988 | 1 | Insect resistant, Disease resistant |
| IRRI | IR74 | 3 | 4.7 | 1988 | 1 | Abiotic stress tolerant, Insect resistant |
| IRRI | PSB Rc1 | 3 | 2.4 | 1990 | 1 | Early maturing, Disease resistant |
| IRRI | PSB Rc2 | 3 | 5.1 | 1991 | 1 | Disease resistant |
| IRRI | PSB Rc4 | 3 | 4.6 | 1991 | 1 | Early maturing, Disease resistant |
| IRRI | PSB Rc10 | 3 | 4.8 | 1992 | 1 | Early maturing, Insect resistant, Pest resistant |
| UPLB | PSB Rc12 | 3 | 3.5 | 1992 | 2 | Early maturing |
| UPLB | PSB Rc14 | 3 | 3.6 | 1992 | 2 | Early maturing, High head rice recovery |
| PhilRice | PSB Rc6 | 3 | 5.2 | 1992 | 2 | Early maturing |
| PhilRice | PSB Rc8 | 3 | 5.0 | 1992 | 2 | Early maturing |
| IRRI | PSB Rc18 | 3 | 5.1 | 1994 | 1 | Moderately insect resistant, Moderately disease resistant |
| IRRI | PSB Rc20 | 3 | 4.9 | 1994 | 1 | Early maturing |
| UPLB | PSB Rc22 | 3 | 5.0 | 1994 | 2 | Moderately insect resistant, Moderately disease resistant |
| PhilRice | PSB Rc24 | 3 | 3.1 | 1994 | 2 | Early maturing |
| IRRI | IR71604 | 3 | 4.0 | 1995 | 1 | Moderate yield advantage |
| IRRI | PSB Rc28 | 3 | 5.0 | 1995 | 1 | Early maturing, Disease resistant |
| IRRI | PSB Rc30 | 3 | 5.0 | 1995 | 1 | Moderately insect resistant, Moderately disease resistant |
| UPLB | PSB Rc32 | 3 | 5.2 | 1995 | 1 | Early maturing, Insect resistant, Disease resistant |
| PhilRice | PSB Rc42 | 3 | 3.2 | 1995 | 2 | Early maturing, Disease resistant |
| IRRI | PSB Rc44 | 3 | 4.1 | 1995 | 1 | Insect resistant, High head rice recovery |
| IRRI | PSB Rc46 | 3 | 4.3 | 1995 | 1 | Moderately disease resistant |
| IRRI | PSB Rc48 | 3 | 2.7 | 1995 | 1 | Abiotic stress tolerant |
| IRRI | PSB Rc50 | 3 | 3.0 | 1995 | 1 | Abiotic stress tolerant, Insect resistant, Disease resistant |
| IRRI | PSB Rc5 | 4 | 2.9 | 1997 | 1 | Disease resistant |
| IRRI | PSB Rc52 | 4 | 5.3 | 1997 | 1 | Early maturing |
| IRRI | PSB Rc54 | 4 | 5.0 | 1997 | 1 | Early maturing, Disease resistant |
| PhilRice | PSB Rc56 | 4 | 5.3 | 1997 | 2 | Early maturing |
| UPLB | PSB Rc58 | 4 | 4.9 | 1997 | 2 | Disease resistant |
| IRRI | PSB Rc60 | 4 | 3.6 | 1997 | 1 | Early maturing |
| PhilRice | PSB Rc62 | 4 | 3.7 | 1997 | 4 | Disease resistant |
| IRRI | PSB Rc64 | 4 | 5.0 | 1997 | 1 | Moderately insect resistant, Moderately disease resistant |
| PhilRice | PSB Rc66 | 4 | 5.2 | 1997 | 2 | Disease resistant |
| IRRI | PSB Rc68 | 4 | 3.4 | 1997 | 2 | Moderately insect resistant, Moderately disease resistant |
| IRRI | PSB Rc70 | 4 | 3.2 | 1997 | 1 | Early maturing |
| IRRI | PSB Rc72H | 4 | 5.4 | 1997 | 1 | Moderately disease resistant |
| UPLB | PSB Rc74 | 4 | 5.2 | 1998 | 1 | Early maturing, Insect resistant |
| IRRI | PSB Rc80 | 4 | 5.0 | 2000 | 1 | Early maturing |
| IRRI | PSB Rc82 | 4 | 5.4 | 2000 | 1 | Early maturing, Disease resistant |
| IRRI | PSB Rc84 | 4 | 2.0 | 2000 | 4 | Abiotic stress tolerant, Early maturing |
| IRRI | PSB Rc86 | 4 | 2.1 | 2000 | 2 | Abiotic stress tolerant, Early maturing |
| IRRI | PSB Rc88 | 4 | 2.2 | 2000 | 1 | Abiotic stress tolerant |
| IRRI | IR69726 | 4 | 4.4 | 2001 | 1 | Moderately insect resistant |
| PhilRice | NSIC Rc104 | 4 | 4.3 | 2001 | 4 | Moderately insect resistant, Moderately disease resistant |
| IRRI | NSIC Rc106 | 4 | 2.9 | 2001 | 2 | Abiotic stress tolerant, Insect resistant |
| PhilRice | NSIC Rc108 | 4 | 2.9 | 2001 | 2 | Abiotic stress tolerant |
| PhilRice | NSIC Rc11 | 4 | 2.6 | 2001 | 4 | Insect resistant |
| IRRI | NSIC Rc9 | 4 | 2.9 | 2001 | 2 | Moderately insect resistant, Moderately disease resistant |
| IRRI | PSB Rc102 | 4 | 2.3 | 2001 | 1 | Moderately insect resistant |
| PhilRice | PSB Rc7 | 4 | 2.9 | 2001 | 2 | Disease resistant |
| PhilRice | PSB Rc90 | 4 | 3.4 | 2001 | 2 | Abiotic stress tolerant, High head rice recovery |
| IRRI | PSB Rc92 | 4 | 3.6 | 2001 | 2 | High head rice recovery |
| IRRI | PSB Rc94 | 4 | 3.3 | 2001 | 1 | High head rice recovery |
| IRRI | PSB Rc96 | 4 | 3.6 | 2001 | 1 | High head rice recovery |
| UPLB | PSB Rc98 | 4 | 2.6 | 2001 | 1 | Insect resistant, High head rice recovery |
| IRRI | IR73885 | 4 | 5.7 | 2002 | 1 | Early maturing |
| IRRI | NSIC Rc110 | 4 | 4.8 | 2002 | 1 | Early maturing, High head rice recovery |
| IRRI | NSIC Rc112 | 4 | 4.9 | 2002 | 1 | Early maturing, High head rice recovery |
| IRRI | NSIC Rc114H | 4 | 5.8 | 2002 | 1 | Early maturing |
| IRRI | NSIC Rc116H | 4 | 5.8 | 2002 | 1 | Early maturing |
| IRRI | NSIC Rc118 | 4 | 4.6 | 2003 | 1 | Early maturing |
| PhilRice | NSIC Rc120 | 4 | 4.8 | 2003 | 2 | Early maturing |
| IRRI | NSIC Rc122 | 4 | 4.7 | 2003 | 1 | Insect resistant, Disease resistant |
| PhilRice | NSIC Rc128 | 4 | 5.5 | 2004 | 4 | High head rice recovery |
| UPLB | NSIC Rc13 | 4 | 4.7 | 2004 | 2 | High head rice recovery, Sticky |
| PhilRice | NSIC Rc15 | 4 | 5.4 | 2004 | 4 | High head rice recovery, Sticky |
| PhilRice | NSIC Rc17 | 4 | 4.2 | 2004 | 4 | Sticky |
| PhilRice | NSIC Rc134 | 4 | 5.4 | 2005 | 3 | Early maturing |
| IRRI | NSIC Rc136H | 4 | 6.7 | 2006 | 1 | Early maturing |
| PhilRice | NSIC Rc138 | 4 | 5.4 | 2006 | 4 | Early maturing |
| IRRI | NSIC Rc140 | 4 | 5.7 | 2006 | 1 | Early maturing |
| PhilRice | NSIC Rc142 | 4 | 5.6 | 2006 | 2 | Early maturing |
| UPLB | NSIC Rc144 | 4 | 5.3 | 2006 | 3 | Early maturing |
| PhilRice | NSIC Rc146 | 4 | 4.6 | 2006 | 4 | Early maturing, Aromatic |
| IRRI | NSIC Rc148 | 4 | 4.6 | 2007 | 1 | Early maturing |
| PhilRice | NSIC Rc150 | 4 | 5.9 | 2007 | 3 | Early maturing |
| PhilRice | NSIC Rc152 | 4 | 6.0 | 2007 | 4 | Early maturing, High head rice recovery |
| PhilRice | NSIC Rc154 | 4 | 5.9 | 2007 | 3 | Early maturing |
| UPLB | NSIC Rc156 | 4 | 5.7 | 2007 | 2 | Early maturing |
| IRRI | NSIC Rc158 | 4 | 6.0 | 2007 | 1 | Early maturing |
| PhilRice | NSIC Rc160 | 4 | 5.6 | 2007 | 2 | Early maturing, High head rice recovery |
| IRRI | NSIC Rc170 | 4 | 4.5 | 2008 | 4 | Early maturing, High head rice recovery |
| IRRI | NSIC Rc172 | 4 | 4.6 | 2008 | 2 | Early maturing |
| IRRI | NSIC Rc182 | 4 | 2.8 | 2009 | 2 | Abiotic stress tolerant, Early maturing |
| PhilRice | NSIC Rc184 | 4 | 3.1 | 2009 | 2 | Abiotic stress tolerant |
| PhilRice | NSIC Rc188 | 4 | 3.2 | 2009 | 3 | Abiotic stress tolerant, High head rice recovery |
| PhilRice | NSIC Rc190 | 4 | 2.9 | 2009 | 2 | Abiotic stress tolerant, High head rice recovery |
| IRRI | NSIC Rc192 | 4 | 3.7 | 2009 | 1 | Abiotic stress tolerant, Early maturing |
| IRRI & PhilRice | NSIC Rc194 | 4 | 2.5 | 2009 | 1 | Abiotic stress tolerant |
| IRRI | NSIC Rc212 | 4 | 6.0 | 2009 | 1 | Extra long grain |
| IRRI | NSIC Rc214 | 4 | 6.0 | 2009 | 1 | Extra long grain |
| IRRI | NSIC Rc220SR | 4 | 2.8 | 2009 | 1 | Early maturing, High head rice recovery |
| IRRI | NSIC Rc222 | 4 | 6.1 | 2009 | 1 | Early maturing |
| PhilRice | NSIC Rc224 | 4 | 5.8 | 2010 | 3 | Early maturing |
| PhilRice | NSIC Rc226 | 4 | 6.2 | 2010 | 2 | Early maturing |
| IRRI | NSIC Rc23 | 4 | 3.0 | 2011 | 2 | Early maturing, Insect resistant, Disease resistant |
| IRRI | NSIC Rc238 | 4 | 6.4 | 2011 | 1 | Early maturing, Insect resistant, Disease resistant |
| PhilRice | NSIC Rc272 | 4 | 3.0 | 2011 | 4 | High yield advantage, Early maturing, Disease resistant, Insect resistant |
| IRRI | NSIC Rc274 | 4 | 3.0 | 2011 | 1 | High yield advantage, Early maturing, Disease resistant, Insect resistant |
| IRRI | NSIC Rc278 | 4 | 2.4 | 2011 | 2 | Early maturing, Disease resistant, Insect resistant |
| IRRI | NSIC Rc280 | 4 | 2.5 | 2011 | 4 | Disease resistant, Aromatic |
| IRRI | NSIC Rc284 | 4 | 3.7 | 2011 | 1 | Early maturing, Disease resistant, Insect resistant, Extra long grain |
| UPLB | NSIC Rc286 | 4 | 3.5 | 2011 | 4 | Early maturing |
| IRRI | NSIC Rc296 | 4 | 3.2 | 2011 | 1 | Abiotic stress tolerant, High yield advantage, High head rice recovery |
| PhilRice | NSIC Rc300 | 4 | 5.7 | 2012 | 3 | Early maturing |
| IRRI | NSIC Rc302 | 4 | 5.7 | 2012 | 1 | Early maturing, Disease resistant, Extra long grain |
| IRRI | NSIC Rc326 | 4 | 2.4 | 2013 | 1 | Abiotic stress tolerant, High yield advantage, Early maturing |
| IRRI | NSIC Rc328 | 4 | 2.4 | 2013 | 1 | Abiotic stress tolerant, High yield advantage, Early maturing |
| IRRI | NSIC Rc334 | 4 | 2.5 | 2013 | 1 | Abiotic stress tolerant, High yield advantage, Early maturing, Extra long grain |
| IRRI | NSIC Rc336 | 4 | 3.0 | 2013 | 1 | Abiotic stress tolerant, High yield advantage, Early maturing |
| IRRI | NSIC Rc340 | 4 | 2.5 | 2013 | 1 | Abiotic stress tolerant, High yield advantage, Early maturing |
| IRRI | NSIC Rc25 | 4 | 3.0 | 2014 | 1 | Early maturing |
| IRRI | NSIC Rc27 | 4 | 2.7 | 2014 | 1 | High yield advantage, Early maturing, Extra long grain |
| IRRI | NSIC Rc29 | 4 | 2.3 | 2014 | 1 | Early maturing |
| IRRI | NSIC Rc352 | 4 | 5.1 | 2014 | 2 | Early maturing |
| IRRI | NSIC Rc356 | 4 | 5.0 | 2014 | 2 | Moderate yield advantage |
| IRRI | NSIC Rc360 | 4 | 5.2 | 2014 | 2 | Moderate yield advantage |
| IRRI | NSIC Rc390 | 4 | 4.0 | 2014 | 1 | Abiotic stress tolerant, High yield advantage, Early maturing |
| IRRI | NSIC Rc392 | 4 | 3.2 | 2014 | 1 | Abiotic stress tolerant, Early maturing, High head rice recovery |
| IRRI | NSIC Rc400 | 4 | 5.8 | 2015 | 1 | High head rice recovery, Slightly aromatic |
| IRRI | NSIC Rc420 | 4 | 3.7 | 2015 | 1 | Early maturing |
| IRRI | NSIC Rc428 | 4 | 3.5 | 2015 | 2 | Early maturing, Disease resistant, High head rice recovery |
| IRRI | NSIC Rc434 | 4 | 3.4 | 2016 | 1 | Early maturing |
| IRRI | NSIC Rc436 | 4 | 5.7 | 2016 | 4 | Early maturing |
| IRRI | NSIC Rc442 | 4 | 6.1 | 2016 | 1 | Early maturing |
| IRRI | NSIC Rc460 | 4 | 4.7 | 2016 | 1 | Early maturing, Insect resistance, High head rice recovery, High zinc |
| IRRI | NSIC Rc464 | 4 | 3.0 | 2016 | 1 | Abiotic stress tolerant, Early maturing |
| IRRI | NSIC Rc468 | 4 | 3.6 | 2016 | 4 | Abiotic stress tolerant, High yield advantage, High head rice recovery |
| IRRI | NSIC Rc478 | 4 | 3.8 | 2016 | 1 | Early maturing |
| IRRI | NSIC Rc480 | 4 | 3.2 | 2016 | 4 | Abiotic stress tolerant, Early maturing |

Note: excludes varieties not found in IRIS database.

Sources: IRIS and PhilRice

Appendix Table A2. IRRI’s contributions to rice varietal development in Bangladesh,1970-2018.

| Institution | Season | Variety name | Generation^^[[3]](#footnote-3)^^ | Ave. Yield (t/ha) | Release year | Origin of variety^^[[4]](#footnote-4)^^ | Main Traits |
| --- | --- | --- | --- | --- | --- | --- | --- |
| BRRI | *Aus, Boro* | BR 1 | 1 | 4.00 (*Aus*), 5.50 (*Boro*) | 1970 | 2 | High yield advantage |
| BRRI | *Aus, Boro* | BR 2 | 1 | 4.00 (*Aus*), 5.00 (*Boro*) | 1971 | 3 | High yield advantage, Disease resistant |
| BRRI | *Aus, Boro* | BR 3 | 1 | 4.00 (*Aus*), 6.50 (*Boro*) | 1973 | 2 | High yield advantage, Disease resistant |
| BRRI | *Aus, Aman* | BR 4 | 2 | 4.50 (*Aus*), 5.00 (*Aman*) | 1975 | 1 | High yield advantage |
| BRRI | *Aus, Boro* | BR 6 | 2 | 3.50 (*Aus*), 4.50 (*Boro*) | 1977 | 1 | Early maturing |
| BRRI | *Aus, Boro* | BR 7 | 2 | 3.50 (*Aus*), 4.50 (*Boro*) | 1977 | 1 | Disease resistant |
| BRRI | *Aus, Boro* | BR 8 | 2 | 5.00 (*Aus*), 6.00 (*Boro*) | 1978 | 1 | High yield advantage, Disease resistant |
| BRRI | *Aus, Boro* | BR 9 | 2 | 5.00 (*Aus*), 6.00 (*Boro*) | 1978 | 1 | High yield advantage |
| BRRI | *Aus* | BR 12 | 2 | 4.50 | 1983 | 2 | Disease resistant |
| BRRI | *Aus* | BR 14 | 2 | 5.00 | 1983 | 2 | High yield advantage, High protein |
| BRRI | *Aus, Boro* | BR 16 | 2 | 5.00 (*Aus*), 6.00 (*Boro*) | 1983 | 1 | High yield advantage |
| BRRI | *Aus, Boro* | BR 20 | 3 | 3.50 (*Aus*), 3.00 (*Boro*) | 1986 | 1 | Early maturing, Disease resistant |
| BRRI | *Aus, Boro* | BR 21 | 3 | 3.00 (*Aus*), 2.50 (*Boro*) | 1986 | 2 | Early maturing, Disease resistant |
| BRRI | *Aus, Boro* | BR 24 | 3 | 3.50 (*Aus*), 3.00 (*Boro*) | 1992 | 2 | Disease resistant |
| BRRI | *Aus, Boro* | BRRI dhan 26 | 3 | 4.00 (*Aus*), 3.50 (*Boro*) | 1993 | 1 | Early maturing, Disease resistant |
| BRRI | *Aus* | BRRI dhan 27 | 3 | 4.00 | 1994 | 2 | Disease resistant |
| BRRI | *Aus, Boro* | BRRI dhan 28 | 3 | 5.50 (*Aus*), 6.00 (*Boro*) | 1994 | 2 | High yield advantage, Early maturing |
| BRRI | *Aus, Boro* | BRRI dhan 29 | 3 | 7.00 (*Aus*), 7.50 (*Boro*) | 1994 | 3 | High yield advantage, Disease resistant |
| BRRI | *Aus, Aman* | BRRI dhan 30 | 3 | 4.00 (*Aus*), 5.00 (*Aman*) | 1994 | 2 | Moderate yield advantage |
| BRRI | *Aus, Aman* | BRRI dhan 42 | 4 | 3.50 (*Aus*) 3.00 (*Aman*) | 2004 | 2 | Abiotic stress tolerant, Early maturing, Disease resistant |
| BRRI | *Aus* | BRRI dhan 43 | 4 | 3.50 | 2004 | 3 | Abiotic stress tolerant, Early maturing, Disease resistant |
| BRRI | *Aus, Boro* | BRRI dhan 48 | 4 | 5.50 (*Aus*), 5.00 (*Boro*) | 2008 | 2 | High yield advantage |
| BRRI | *Aus, Boro* | BRRI dhan 50 | 4 | 5.50 (*Aus*), 6.00 (*Boro*) | 2008 | 2 | High yield advantage, Aromatic |
| BRRI | *Aus, Boro* | BRRI dhan 55 | 4 | 5.00 (*Aus*), 7.00 (*Boro*) | 2011 | 2 | Abiotic stress tolerant, High yield advantage, Early maturing |
| BINA | *Aus, Boro* | Iratom 24 | 2 | 3.50 (*Aus*), 6.50 (*Boro*) | 1975 | 2 | Early maturing |
| BRRI | *Boro* | BR 12 | 2 | 5.50 | 1983 | 2 | High yield advantage, Disease resistant |
| BRRI | *Boro* | BR 14 | 2 | 6.00 | 1983 | 2 | High yield advantage |
| BRRI | *Boro* | BR 15 | 2 | 5.50 | 1983 | 1 | Moderate yield advantage |
| BRRI | *Boro* | BR 17 | 3 | 6.00 | 1985 | 2 | High yield advantage |
| BRRI | *Boro* | BR 18 | 3 | 6.00 | 1985 | 2 | High yield advantage |
| BRRI | *Boro* | BR 19 | 3 | 6.00 | 1985 | 1 | High yield advantage, Disease resistant |
| BRRI | *Boro, Aman* | BRRI dhan 32 | 3 | 4.50 (*Boro*), 5.00 (*Aman*) | 1994 | 3 | Early maturing, Disease resistant |
| BRRI | *Boro, Aman* | BRRI dhan 33 | 4 | 4.00 (*Boro*), 4.50 (*Aman*) | 1997 | 3 | Early maturing, Insect resistant, Disease resistant |
| BRRI | *Boro* | BRRI dhan 35 | 4 | 5.00 | 1998 | 2 | Disease resistant |
| BRRI | *Boro* | BRRI dhan 36 | 4 | 5.00 | 1998 | 1 | Abiotic stress tolerant, Disease resistant |
| BRRI | *Boro* | BRRI dhan 45 | 4 | 6.50 | 2005 | 2 | Abiotic stress tolerant, High yield advantage, Disease resistant |
| BRRI | *Boro, Aman* | BRRI dhan 46 | 4 | 4.00 (*Boro*), 4.70 (*Aman*) | 2007 | 2 | Moderate yield advantage |
| BRRI | *Boro* | BRRI dhan 47 | 4 | 6.00 | 2007 | 2 | Abiotic stress tolerant, High yield advantage |
| BRRI | *Boro* | BRRI dhan 58 | 4 | 7.20 | 2012 | 3 | High yield advantage, Disease resistant |
| BRRI | *Boro* | BRRI dhan 59 | 4 | 7.10 | 2013 | 2 | High yield advantage, Lodging tolerant |
| BRRI | *Boro* | BRRI dhan 60 | 4 | 7.30 | 2013 | 3 | High yield advantage |
| BRRI | *Boro* | BRRI dhan 61 | 4 | 6.30 | 2013 | 2 | Abiotic stress tolerant, High yield advantage |
| BRRI | *Boro* | BRRI dhan 63 | 4 | 7.00 | 2014 | 3 | High yield advantage |
| BRRI | *Boro* | BRRI dhan 64 | 4 | 6.50 | 2014 | 2 | High yield advantage, High zinc |
| BRRI | *Boro* | BRRI dhan 67 | 4 | 6.00 | 2014 | 2 | Abiotic stress tolerant, High yield advantage |
| BRRI | *Boro* | BRRI dhan 69 | 4 | 7.30 | 2014 | 3 | High yield advantage, Lodging tolerant |
| BRRI | *Boro* | BRRI dhan 74 | 4 | 7.10 | 2015 | 2 | High yield advantage, Lodging tolerant, High zinc |
| BRRI | *Boro* | BRRI dhan 81 | 4 | 6.50 | 2017 | 3 | High yield advantage, Lodging tolerant, High protein |
| BRRI | *Boro* | BRRI dhan 84 | 4 | 6.50 | 2017 | 2 | High yield advantage, High zinc |
| BRRI | *Boro* | BRRI dhan 88 | 4 | 7.00 | 2018 | 3 | High yield advantage, Disease resistant, High protein |
| BRRI | *Boro* | BRRI dhan 89 | 4 | 8.00 | 2018 | 2 | High yield advantage, High protein |
| BRRI | *Boro* | BRRI dhan 92 | 4 | 8.40 | 2019 | 2 | High yield advantage |
| BINA | *Boro, Aman* | BINA dhan 4 | 4 | 4.00 (*Boro*), 4.70 (*Aman*) | 1998 | 3 | Early maturing, Insect resistant, Disease resistant |
| BINA | *Boro* | BINA dhan 5 | 4 | 7.00 | 1998 | 3 | High yield advantage, Lodging tolerant |
| BINA | *Boro* | BINA dhan 6 | 4 | 7.50 | 1998 | 3 | High yield advantage |
| BINA | *Boro* | BINA dhan 10 | 4 | 7.00 | 2011 | 2 | Abiotic stress tolerant, High yield advantage, Early maturing |
| BAU | *Boro* | BAU dhan 3 | 4 | 7.10 | 2019 | 1 | Abiotic stress tolerant, High yield advantage, Early maturing, Disease resistant |
| BRRI | *Aman* | BR 10 | 2 | 5.50 | 1980 | 1 | High yield advantage, Insect resistant, Disease resistant |
| BRRI | *Aman* | BR 11 | 2 | 5.50 | 1980 | 1 | High yield advantage, Insect resistant, Disease resistant |
| BRRI | *Aman* | BR 22 | 3 | 5.00 | 1988 | 3 | High yield advantage, Insect resistant, Disease resistant |
| BRRI | *Aman* | BR 23 | 3 | 5.50 | 1988 | 3 | High yield advantage, Insect resistant, Disease resistant |
| BRRI | *Aman* | BR 25 | 3 | 4.50 | 1992 | 2 | Insect resistant, Disease resistant |
| BRRI | *Aman* | BRRI dhan 31 | 3 | 5.00 | 1994 | 2 | High yield advantage, Early maturing, Insect resistant, Disease resistant |
| BRRI | *Aman* | BRRI dhan 34 | 4 | 3.50 | 1997 | 4 | Aromatic |
| BRRI | *Aman* | BRRI dhan 39 | 4 | 4.50 | 1999 | 4 | Early maturing |
| BRRI | *Aman* | BRRI dhan 40 | 4 | 4.50 | 2003 | 2 | Abiotic stress tolerant |
| BRRI | *Aman* | BRRI dhan 41 | 4 | 4.50 | 2003 | 3 | Abiotic stress tolerant |
| BRRI | *Aman* | BRRI dhan 44 | 4 | 5.50 | 2005 | 3 | High yield advantage, Disease resistant |
| BRRI | *Aman* | BRRI dhan 49 | 4 | 5.50 | 2008 | 2 | High yield advantage |
| BRRI | *Aman* | BRRI dhan 51 | 4 | 4.50 | 2010 | 2 | Abiotic stress tolerant |
| BRRI | *Aman* | BRRI dhan 52 | 4 | 5.00 | 2010 | 2 | Abiotic stress tolerant, High yield advantage |
| BRRI | *Aman* | BRRI dhan 53 | 4 | 4.50 | 2010 | 3 | Abiotic stress tolerant |
| BRRI | *Aman* | BRRI dhan 56 | 4 | 4.50 | 2011 | 2 | Abiotic stress tolerant, Early maturing |
| BRRI | *Aman* | BRRI dhan 57 | 4 | 4.00 | 2011 | 3 | Abiotic stress tolerant, Early maturing |
| BRRI | *Aman* | BRRI dhan 66 | 4 | 4.50 | 2014 | 1 | Abiotic stress tolerant, Early maturing |
| BRRI | *Aman* | BRRI dhan 70 | 4 | 5.00 | 2015 | 1 | High yield advantage, Aromatic |
| BRRI | *Aman* | BRRI dhan 71 | 4 | 5.50 | 2015 | 1 | Abiotic stress tolerant, High yield advantage, Early maturing |
| BRRI | *Aman* | BRRI dhan 72 | 4 | 6.00 | 2015 |  | High yield advantage, High zinc |
| BRRI | *Aman* | BRRI dhan 73 | 4 | 4.75 | 2015 | 2 | Abiotic stress tolerant |
| BRRI | *Aman* | BRRI dhan 75 | 4 | 5.50 | 2016 | 2 | High yield advantage, Early maturing, High protein |
| BRRI | *Aman* | BRRI dhan 76 | 4 | 5.00 | 2016 | 2 | Abiotic stress tolerant, High yield advantage |
| BRRI | *Aman* | BRRI dhan 77 | 4 | 5.00 | 2016 | 2 | Abiotic stress tolerant, High yield advantage |
| BRRI | *Aman* | BRRI dhan 78 | 4 | 4.50 | 2016 | 1 | Abiotic stress tolerant |
| BRRI | *Aman* | BRRI dhan 79 | 4 | 5.50 | 2017 | 3 | Abiotic stress tolerant, High yield advantage |
| BRRI | *Aman* | BRRI dhan 80 | 4 | 5.00 | 2017 | 1 | High yield advantage |
| BRRI | *Aman* | BRRI dhan 87 | 4 | 6.50 | 2018 | 3 | High yield advantage |
| BRRI | *Aman* | BRRI dhan 91 | 4 | 3.50 | 2019 | 3 | Abiotic stress tolerant |
| BRRI | *Aman* | BRRI HV4 | 4 | 6.50 | 2010 | 2 | High yield advantage |
| BRRI | *Aman* | BRRI HV6 | 4 | 6.50 | 2017 | 2 | High yield advantage, Early maturing, Disease resistant |
| BINA | *Aman* | BINA dhan 8 | 4 | 5.00 | 2010 | 2 | High yield advantage, Early maturing |
| BINA | *Aman* | BINA dhan 11 | 4 | 5.00 | 2013 | 2 | Abiotic stress tolerant, High yield advantage, Early maturing |
| BINA | *Aman* | BINA dhan 12 | 4 | 4.25 | 2013 | 2 | Abiotic stress tolerant, Early maturing |
| BINA | *Aman* | BINA dhan 15 | 4 | 4.75 | 2014 | 1 | Early maturing |
| BINA | *Aman* | BINA dhan 16 | 4 | 5.60 | 2014 | 3 | High yield advantage, Early maturing |
| BINA | *Aman* | BINA dhan 17 | 4 | 6.80 | 2016 | 3 | High yield advantage |
| RU | *Aman* | RU dhan 1 | 4 | 6.20 | 2019 | 1 | High yield advantage, Disease resistant |

Sources: BRRI, BINA, and DAE

Appendix Figure A1. The patterns of adoption of leading rice varieties in the Philippines based on the geometric rule, 1990 to 2018.

Appendix Figure A2.1. The patterns of adoption of leading rice varieties for the *Boro* season in Bangladesh based on the geometric rule, 1990 to 2018.

Appendix Figure A2.2. The patterns of adoption of leading rice varieties for the *Aman* season in Bangladesh based on the geometric rule, 1990 to 2018.

Appendix Figure A2.3. The patterns of adoption of leading rice varieties for the *Aus* season in Bangladesh based on the geometric rule, 1990 to 2018.

Appendix Figure A3.1. Discounted total benefits and costs (million USD; 5%; t=0 in 1990) in the Philippines based on two attribution rules, 1990 to 2018. Appendix Figure A3.2. Discounted total benefits and costs (million USD; 5%; t=0 in 1990) in Bangladesh based on geometric rule, 1990 to 2018.

1. 1 indicates first-generation improved varieties released before 1975; 2 indicates second-generation released from 1975 to 1984; 3 indicates third-generation released from 1985 to 1995; 4 indicates fourth-generation released after 1995 [↑](#footnote-ref-1)
2. 1 indicates two IRRI lines as parents; 2 indicates 1 IRRI line as parent; 3 indicates with other IRRI ancestry; 4 indicates without IRRI connection [↑](#footnote-ref-2)
3. 1 indicates first-generation improved varieties released before 1975; 2 indicates second-generation released from 1975 to 1984; 3 indicates third-generation released from 1985 to 1995; 4 indicates fourth-generation released after 1995 [↑](#footnote-ref-3)
4. 1 indicates two IRRI lines as parents; 2 indicates 1 IRRI line as parent; 3 indicates with other IRRI ancestry; 4 indicates without IRRI connection [↑](#footnote-ref-4)
